# Phosphate sensing by PhoPR regulates the cytotoxicity of Staphylococcus aureus

**DOI:** 10.1099/mic.0.001606

**Published:** 2025-09-02

**Authors:** Nathanael Palk, Tarcisio Brignoli, Marcia Boura, Ruth C. Massey

**Affiliations:** 1 School of Cellular and Molecular Medicine, University of Bristol, Bristol, UK; 2 Department of Biosciences, Università degli Studi di Milano, Milan, Italy; 3 School of Microbiology, UCC, Cork, Ireland; 4 APC Microbiome Ireland, UCC, Cork, Ireland

**Keywords:** cytolytic toxins, cytotoxicity, phenol soluble modulins (PSMs), PhoPR, *Staphylococcus aureus*, two-component systems

## Abstract

*Staphylococcus aureus* has evolved a complex regulatory network to coordinate expression of virulence factors, including cytolytic toxins, with host environmental signals. Central to this network are two-component systems (TCSs), in which a histidine kinase senses an external signal and activates a response regulator via phosphorylation, leading to changes in gene expression. Using a comprehensive screen of transposon mutants in each of the non-essential histidine kinase and response regulator genes in *S. aureus*, we demonstrate that 11 of these 16 systems regulate cytotoxicity. Further characterization of the phosphate-sensing PhoPR system revealed that PhoP affects cytotoxicity in a manner mediated through the Agr quorum-sensing system. Notably, we found that unphosphorylated PhoP is an activator of Agr activity, whilst phosphorylated PhoP also acts as a weak activator of Agr activity in high-phosphate environments but as a repressor in low-phosphate environments. Furthermore, overexpression of PhoP resulted in upregulation of *α*-type phenol-soluble modulins, which may also contribute to the cytotoxicity phenotype observed in the *phoP* mutant. Overall, we have demonstrated that phosphate sensing through PhoPR is a novel regulator of cytotoxicity in *S. aureus*. Moreover, our study challenges the canonical model of TCSs as simple on/off systems and highlights the importance of unphosphorylated response regulators in gene regulation.

## Importance

The production of cytolytic toxins is the major means by which bacterial pathogens damage host tissue and cause disease. Understanding the activity and regulation of these toxins is critical for the identification of means to block them and prevent the development of disease. In this study, we focused on a specific regulatory mechanism, the two-component systems (TCSs), that enable bacteria to sense their environment and adapt accordingly. In the traditional model of a TCS, a response regulator (RR) is phosphorylated by a histidine kinase (HK), which enables it to activate or repress expression of target genes, including toxins or regulators of toxins. We found that 11 of *S. aureus*’ 16 TCSs affect toxin production, highlighting that *S. aureus* integrates a broad range of environmental cues to regulate toxicity. We focused on one of these TCSs, the PhoPR system, and found that sensing of inorganic phosphate is a novel regulator of cytotoxicity in *S. aureus*. Furthermore, we found that the RR of this system acts as a strong activator of cytotoxicity in its unphosphorylated form, challenging the traditional model of a TCS as only active upon signal activation.

## Introduction

*Staphylococcus aureus* is a Gram-positive bacterium and opportunistic human pathogen which was associated with more than one million deaths globally in 2019 [1]. It is a leading cause of a wide range of infections, including skin and soft tissue infections, bacteraemia, endocarditis, pneumonia and osteomyelitis [2]. The ability of *S. aureus* to cause a diverse spectrum of diseases is generally attributed to an impressive arsenal of virulence factors [3,6]. These include several cytolytic toxins, which lyse host cells by forming pores or channels in their membranes, referred to from herein as cytotoxicity. Alpha-haemolysin (Hla), the bicomponent leukotoxins (LukSF-PV, LukAB and LukED) and gamma haemolysin (HlgAB and HlgCB) use a receptor-dependent mechanism to cause lysis. In contrast, phenol-soluble modulins (PSMs) have detergent-like properties [7,9]. These have been linked to several aspects of *S. aureus* pathogenesis, including immune evasion, nutrient acquisition and inflammation [8,10, 11]. Importantly, cytolytic toxins are expressed in coordination with host environmental signals through a complex regulatory network, and understanding this is key to linking their pathogenic role to specific disease states [12,14].

In bacteria, TCSs are a widespread mechanism used to sense and respond to environmental signals [15]. In response to a specific signal, a membrane-bound HK will undergo ATP-dependent autophosphorylation. Subsequently, the phosphoryl group will be transferred to a cytoplasmic DNA-binding RR [16]. Phosphorylation of the RR induces a conformation change and modulates its affinity for gene promoters, where they can act as transcriptional activators or repressors [17]. Some TCSs also contain auxiliary proteins which can be involved in sensing or signal transduction [18,19].

The genome of *S. aureus* contains 16 TCSs, with several of these linked to regulating expression of cytolytic toxins. For example, the accessory gene regulator (Agr) TCS uses quorum sensing to upregulate most *S. aureus* toxins, including Hla, leukotoxins, delta-haemolysin (Hld) and PSMs [20,22]. The staphylococcal accessory element (Sae) TCS responds to phagocytosis-related signals to upregulate expression of Hla, leukotoxins, HlgAB and HlgCB [23,26]. Similarly, the autolysis-related locus (Arl) TCS acts as a metabolic sensor and induces expression of leukotoxins in response to nutritional immunity [13,27,29]. The remaining TCSs have been linked to cell wall biosynthesis and antibiotic resistance (VraRS, GraRS and NsaRS), autolysis (WalRK and LytRS), respiration (SrrBA, AirRS and NreCB) and nutrient sensing (HssRS, KdpDE and PhoPR) [17]. Additionally, there is a TCS with a currently unknown function (DesRK). Each TCS regulates a set of target genes, known as its regulon, and this can include other regulatory elements. For example, the Arl regulon shares ~70% overlap with that of the global regulator MgrA [28]. Moreover, TCSs can affect the activity of each other. For instance, expression of a constitutively active WalR variant has been shown to induce toxin expression through activation of the Sae TCS [30]. The extent of our knowledge on the complex virulence regulatory network in *S. aureus* has been the subject of several reviews [12,31, 32].

Current knowledge of the TCSs which regulate cytotoxicity in *S. aureus* is mainly derived from transcriptional comparisons between wild-type strains and mutant pairs in HK and/or RR genes. However, we are currently lacking a comprehensive study which evaluates and compares the contribution of all the TCSs in *S. aureus* to a single phenotype such as cytotoxicity. To address this, we screened the cytotoxicity of transposon mutants in each non-essential HK and RR gene in the *S. aureus* NTML reference CA-MRSA strain JE2. Using this approach, we identified several TCS mutants with reduced cytotoxicity, including a mutant of the *phoP* gene, the RR from the PhoPR TCS. The *phoP* mutant has a lower abundance of PSMs in the bacterial supernatant due to regulatory control of RNAIII and *α*-type PSMs. Interestingly, we found that PhoP regulation of Agr activity and cytotoxicity is differential depending on the concentration of inorganic phosphate (Pi) in growth media. Furthermore, we show that the phosphorylation state of PhoP is a key factor in mediating this differential regulation.

## Methods

### Bacterial strains and growth conditions

A list of the *S. aureus* strains, plasmids and primers used in this study is available in Tables1^3^. *S. aureus* strains were grown at 37 °C on tryptic soy agar (TSA) or in tryptic soy broth (TSB) with shaking at 180 r.p.m. Mutant strains of all HK and RR genes in the genome of USA300 JE2 were obtained from the Nebraska Transposon Mutant Library (NTML) [33] and grown in media supplemented with erythromycin (5 µg ml^−1^). *S. aureus* strains carrying the pRMC2 plasmid were selected with chloramphenicol (10 µg ml^−1^) and expression of the inserted gene induced with anhydrotetracycline (aTC) (200 ng ml^−1^).

**Table 1.** Strains used in this study

Strain	Description	Reference
JE2	USA300; CA-MRSA, type IV SCCmec; lacking plasmids p01 and p03; wild-type strain of the NTML	[33]
*agrA::tn*	*agrA* transposon mutant in JE2	[33]
*agrC::tn*	*agrC* transposon mutant in JE2	[33]
*saeR::tn*	*saeR* transposon mutant in JE2	[33]
*saeS::tn*	*saeS* transposon mutant in JE2	[33]
*arlR::tn*	*arlR* transposon mutant in JE2	[33]
*arlS::tn*	*arlS* transposon mutant in JE2	[33]
*graR::tn*	*graR* transposon mutant in JE2	[33]
*graS::tn*	*graS* transposon mutant in JE2	[33]
*vraR::tn*	*vraR* transposon mutant in JE2	[33]
*vraS::tn*	*vraS* transposon mutant in JE2	[33]
*nsaR::tn*	*nsaR* transposon mutant in JE2	[33]
*nsaS::tn*	*nsaS* transposon mutant in JE2	[33]
*lytR::tn*	*lytR* transposon mutant in JE2	[33]
*lytS::tn*	*lytS* transposon mutant in JE2	[33]
*kdpE::tn*	*kdpE* transposon mutant in JE2	[33]
*kdpD::tn*	*kdpD* transposon mutant in JE2	[33]
*phoP::tn*	*phoP* transposon mutant in JE2	[33]
*phoR::tn*	*phoR* transposon mutant in JE2	[33]
*hptR::tn*	*hptR* transposon mutant in JE2	[33]
*hptS::tn*	*hptS* transposon mutant in JE2	[33]
*hssR::tn*	*hssR* transposon mutant in JE2	[33]
*hssS::tn*	*hssS* transposon mutant in JE2	[33]
*srrA::tn*	*srrA* transposon mutant in JE2	[33]
*srrB::tn*	*srrB* transposon mutant in JE2	[33]
*nreC::tn*	*nreC* transposon mutant in JE2	[33]
*nreB::tn*	*nreB* transposon mutant in JE2	[33]
*airS::tn*	*airS* transposon mutant in JE2	[33]
*desR::tn*	*desR* transposon mutant in JE2	[33]
*desk::tn*	*desK* transposon mutant in JE2	[33]
*phoP::tn* pRMC2	*phoP* transposon mutant in JE2 transformed with empty pRMC2 vector	This study
*phoP::tn pphoP*	*phoP* transposon mutant complemented with *phoP* gene cloned into the pRMC2 expression plasmid	This study
JE2 pRNAIII*::gfp*	JE2 transformed with GFP reporter plasmid of RNAIII expression	This study
*phoP::tn* pRNAIII*::gfp*	*phoP* transposon mutant transformed with GFP reporter plasmid of RNAIII expression	This study
*agrA::tn* pRNAIII*::gfp*	*agrA* transposon mutant transformed with GFP reporter plasmid of RNAIII expression	This study
JE2 pAH5E	JE2 transformed with YFP reporter vector with no promoter driving *yfp* expression	This study
JE2 p*pstS::yfp*	JE2 transformed with YFP reporter plasmid of *pstS* expression	This study
*phoP::tn* pAH5E	*phoP* transposon mutant transformed with YFP reporter vector with no promoter driving yfp expression	This study
*phoP::tn* p*pstS::yfp*	*phoP* transposon mutant transformed with YFP reporter plasmid of *pstS* expression	This study
*phoP::tn pphoP* (D53A)	*phoP* transposon mutant complemented with *phoP* (D53A mutation) cloned into the pRMC2 expression plasmid	This study
*phoP::tn pphoP* (D53E)	*phoP* transposon mutant complemented with *phoP* (D53E mutation) cloned into the pRMC2 expression plasmid	This study

**Table 2.** Plasmids used in this study

Plasmid	Description	Reference
pRMC2	aTC-inducible expression vector	[67]
*pphoP*	*phoP* gene cloned into the pRMC2 plasmid	This study
*p*RNAIII*::gfp*	GFP reporter plasmid of RNAIII expression	[68]
pAH5E	YFP reporter vector with no promoter driving yfp expression	[37]
p*pstS::yfp*	YFP reporter plasmid of *pstS* expression	[37]
*pphoP* (D53A)	*phoP* (D53A mutation) gene cloned into the pRMC2 plasmid	This study
*pphoP* (D53E)	*phoP* (D53E mutation) gene cloned into the pRMC2 plasmid	This study

**Table 3.** Primers used in this study

Primer	Sequence (5′–3′)
*phoP* F	atatatggtacctaggatgttatttttataaatagttcgg
*phoP* R	atatatgagctcctgcttatcaataacattaagcg
*phoP* D53A F	gatttaattattttagcagttatgctacctaaa
*phoP* D53A R	tttaggtagcataactgctaaaataattaaatc
*phoP* D53E F	gatttaattattttagaagttatgctacctaaa
*phoP* D53E R	tttaggtagcataacttctaaaataattaaatc
RNAIII F	ggaaggagtgatttcaatgg
RNAIII R	gtgaatttgttcactgtgtcg
*pstS* F	gtaatggtggcagtggtaatag
*pstS* R	catccgagtgatcttgagc
*gyrB* F	ggtgactgcattgtcagatgtaaac
*gyrB* R	ctgcttctaaaccttctaatacttgtatttg
*psmα1* F	atgggtatcatcgctgg
*psmα1* R	ttatttaccagtgaattgttcg
*psmα2* F	gggtatcattgcaggaatc
*psmα2* R	ccagtgaatttctcaattaatcc
*psmα3* F	atggaattcgtagcaaaattattc
*psmα3* R	gttgttacctaaaaatttaccaag
*psmα4* F	ggctattgtaggtactatcattaaaatc
*psmα4* R	ttattttgcgaaaatgtcg
*agrA* F	gaggtgcttgagcaagc
*agrA* R	caactgggtcatgcttacg

### THP-1 cytotoxicity assay

THP-1 cells were exposed to the supernatant from *S. aureus* overnight cultures as a measure of cytotoxicity, as they are sensitive to lysis by the widest range of *S. aureus* toxins, including PVL, Hla, Hld and *α*-type PSMs [34,36]. THP-1 cells were sub-cultured every 2–3 days in RPMI 1640 supplemented with FBS (10%), l-glutamine (1 µM), penicillin (200 U ml^−1^) and streptomycin (0.1 mg ml^−1^) at 37 °C with 5% CO2. For the toxicity assay, cells were harvested by centrifugation (1,250 r.p.m., 20 °C, 5 min) and resuspended in Hanks’ Balanced Salt Solution (BioWhittaker) to a final density of 1×10^6^ to 1.5×10^6^ cells ml^−1^. Bacterial supernatants were extracted after 18 h of growth by centrifugation (4,000 r.p.m., 4 °C, 5 min) and incubated with harvested THP-1 cells for 12 min at 37 °C. Cell death was quantified via trypan blue exclusion with percentage (%) THP-1 killing defined as the number of dead THP-1 cells out of the total number of THP-1 cells counted for each sample. Each assay was performed with three technical replicates and three biological replicates. For comparisons across multiple assays, this percentage was taken relative to the THP-1 killing of the wild-type strain (JE2) in each assay to account for any variability in the sensitivity of the THP-1 cells. Incubation of THP-1 cells with TSB was used as a negative control to ensure appropriate viability of the THP-1 cells for each assay.

### Complementation of the *phoP::tn* mutant

To verify that the *phoP::tn* mutation was responsible for the reduced cytotoxicity phenotype, the wild-type *phoP* gene was reintroduced into the transposon mutant via the pRMC2 vector. This vector was selected as it controls gene expression through an aTC-inducible promoter.

A wild-type copy of the *phoP* gene was PCR-amplified from JE2 genomic DNA with KpnI and SacI restriction sites using *phoP* F and *phoP* R primers (Table 3). The PCR product was checked for a product of the correct size by agarose gel electrophoresis and purified using the QIAquick^®^ PCR purification kit (Qiagen). Purified *phoP* and pRMC2 vector were then digested with KpnI and SacI (NEB) restriction enzymes, and the products were purified again. Insert and plasmid DNA were ligated in a 3 : 1 molar ratio using T4 DNA ligase (NEB) to generate p*phoP*. This was transformed into *Escherichia coli* Mach 1 competent cells via heat shock, and successful transformants were confirmed by colony PCR. Plasmids were isolated from Mach 1, passaged through *S. aureus* RN4220 and then transformed into *phoP::tn* via electroporation. For electroporation, competent cells were generated by culturing strains to an OD_600nm_ of 0.5 and washing three times in 0.5 M sucrose. For transformations, competent cells were incubated in ice with 100 ng of plasmid DNA for 30 min. Cells were transferred into 0.2 mm electroporation cuvettes (Bio-Rad) for electroporation (25 µF, 2.5 KV and 100 Ω; pulse time of 2.5 msec). Following electroporation, 700 µl of TSB was added, and cells were incubated at 37 °C for 1 h. Successful transformants were selected for by plating onto TSA with 10 µg ml^−1^ chloramphenicol. To test for complementation of cytotoxicity, strains carrying the pRMC2 vector were cultured in TSB with 10 µg ml^−1^ chloramphenicol and 200 ng ml^−1^ aTC, and supernatants were extracted after 18 h of growth. Cytotoxicity in *phoP::tn* p*phoP* was compared to a strain carrying the empty vector (*phoP::tn* pRMC2), as these could be grown under the same conditions.

### PSM abundance

To extract the supernatant, 1 ml of *S. aureus* overnight culture was centrifuged (13,000 r.p.m., 5 min). The supernatant was then diluted twofold in TSB and combined with 5× protein loading dye (National Diagnostics). Samples were boiled at 98 °C for 5 min and loaded onto 4–12% nUView Tris-Glycine Precast Gels. Protein was separated at 100–130 V until the dye front had run off the gel. Protein bands were visualized using Quick Coomassie Stain (Protein Ark) overnight, and gels were destained in deionized water for at least 5 h. Band intensities were quantified using ImageJ.

### GFP/YFP reporter assays

Reporter plasmids (pRNAIII::*gfp*, pAH5E or p*pstS::yfp*) were transformed into JE2, *phoP::tn* or *agrA::tn*, via electroporation. To determine RNAIII expression in TSB, strains with pRNAIII::*gfp* were grown overnight in TSB with 10 µg ml^−1^ chloramphenicol and normalized to an OD_600nm_ of 0.05 in fresh TSB. GFP fluorescence (485 nm excitation/520 nm emission/1,000 gain) and OD_600nm_ readings were taken every 30 min for a 24 h period with 200 r.p.m. shaking in between readings. Measurements were taken in a black 96-well plate (Costar) using a CLARIOstar plate reader. GFP fluorescence (485 nm excitation/520 nm emission/1,500 gain) and these were normalized to OD_600nm_ readings (F/OD).

To determine RNAIII and *pstS* expression under different phosphate concentrations, overnight cultures of strains carrying either *p*RNAIII*::gfp* or *ppstS::yfp* were washed and resuspended in phosphate-depleted RPMI media (MP Biomedicals). Strains were then normalized to an OD_600nm_ of 0.05 in phosphate-depleted RPMI media with phosphate levels adjusted using 0.2 M sodium phosphate buffer, pH 7.4 (Thermo Fisher), and incubated at 37 °C overnight with shaking. A 200 µl volume of culture was aliquoted into a black 96-well plate (Costar). YFP measurements (485 nm excitation/520 nm emission/1,500 gain) were normalized to OD_600nm_, and the F/OD of a corresponding strain carrying empty pAH5E was subtracted from this value as described previously [37].

### Site-directed mutagenesis

Site-directed mutagenesis was performed to mutate wild-type *phoP* in p*phoP* to two variants: D53A and D53E. PCR amplification was performed using p*phoP* from Mach 1 *E. coli* as template DNA with *phoP* D53A F and *phoP* D53A R or *phoP* D53E F and *phoP* D53E R primers (Table 3). The PCR product was checked for a band of the correct size by agarose gel electrophoresis. One microlitre of DpnI was then added, and the PCR product was incubated at 37 °C for 1 h to remove template DNA. Five microlitres of the product were then transformed into Mach 1, passaged through *S. aureus* RN4220 and then transformed into *phoP::tn* via electroporation.

### mRNA extraction and qRT-PCR

*S. aureus* strains were grown to an OD_600nm_ of 4 in 50 ml TSB and incubated with 200 ng ml^−1^ aTC for 1 h at 37 °C with shaking at 180 r.p.m. Two millilitres of culture were then collected, and RNA was stabilized using RNAprotect bacterial reagent (Qiagen). RNA extractions were performed as described previously [38] using the Quick-RNA fungal/bacterial miniprep kit (Zymo Research). Genomic DNA was removed from RNA samples using a TURBO DNA-free kit (Thermo Fisher), and reverse transcription was performed using the qScript cDNA synthesis kit (Quantabio). To verify that samples were free from DNA contamination, a reverse transcription negative reaction was performed on all RNA samples, and quantitative reverse transcription PCR (qRT-PCR) was performed. Threshold values were then compared to reverse transcription-positive samples. Real-time PCR was performed using a KAPA SYBR fast qPCR kit (Kapa Biosystems), using RNAIII_F and RNAIII_R, *pstS*_F and *pstS*_R or *gyrB*_F and *gyrB*_R. *gyrB* was used as the housekeeping gene. All primer sets were validated using fivefold dilutions of JE2 genomic DNA with efficiencies of 104.19, 106.19% and 100.67% for *pstS*, RNAIII and *gyrB*, respectively.

### Statistical analysis

Statistical comparisons between two samples were performed with an unpaired two-tailed t-test using GraphPad Prism. A *P*-value of <0.05 was considered significant. In our screening of cytotoxicity, multiple *t*-tests were used, and a false discovery rate (FDR) of 1% was applied to account for multiple comparisons. Therefore, samples were considered significant if they had an adjusted *P*-value>0.01. Data are displayed as the mean±sd of the mean, and experiments were performed with three biological replicates.

## Results

### Cytotoxicity is altered in transposon mutants of many TCSs in *S. aureus*

**Fig. 1.**
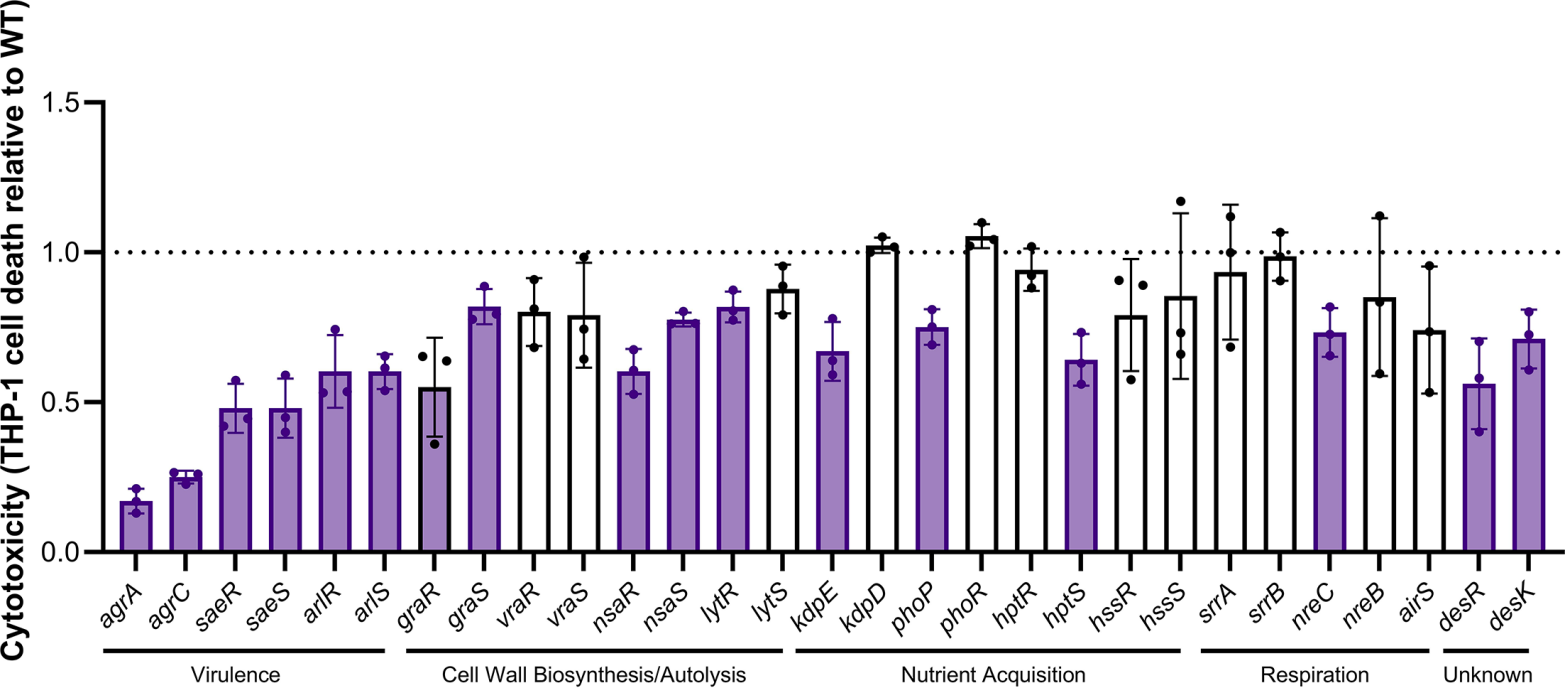
Cytotoxicity is altered in transposon mutants of TCSs in *S. aureus*. To quantify cytotoxicity, the bacterial supernatant from transposon mutants in each non-essential HK and RR gene was incubated with THP-1 cells, and the percentage of dead cells was quantified using trypan blue exclusion. THP-1 killing was calculated relative to the THP-1 killing of JE2 (WT) in each assay. Each dot represents one biological replicate (*n* = 3), error bars represent the sd and statistical significance was determined using multiple *t*-tests with an FDR of 1% applied. Significant hits, which had an adjusted *P*-value ≤0.01, are highlighted in purple.

To identify the TCSs in *S. aureus* that regulate cytotoxicity, we used transposon insertion mutants in the HK and RR genes in the genome of *S. aureus* JE2 from the NTML [33]. As the WalKR TCS is essential for cell viability, there was no mutant strain of the *walK* and *walR* genes available. Additionally, there was no mutant strain of the *airR* gene in the NTML. We tested cytotoxicity using THP-1 cells as these are susceptible to a wide range of *S. aureus* toxins, including PVL, Hla, Hld and *α*-type PSMs [35,36]. From a screening of 29 transposon mutants, 17 demonstrated a statistically significant difference in cytotoxicity compared to the wild-type strain (Fig. 1). All unadjusted and adjusted *P*-values are provided in Table S1 (available in the online Supplementary Material). Importantly, all transposon mutants in TCSs which have been demonstrated in previous studies to directly regulate expression of cytolytic toxins (*agrA::tn*, *agrC::tn*, *saeR::tn*, *saeS::tn*, *arlR::tn* and *arlS::tn*) had a significant reduction in THP-1 killing, providing proof of principle of our cytotoxicity assay [3,12, 28]. The *agrA::tn* and *agrC::tn* mutants had the lowest cytotoxicity to THP-1 cells, confirming previous reports that the Agr system is the major virulence regulator in *S. aureus* JE2 [12,39]. In addition to the established cytotoxicity-linked TCSs, others involved in cell wall biosynthesis and autolysis (*graR::tn*, *graS::tn*, *nsaR::tn*, *nsaS::tn* and *lytR::tn*) had reduced cytotoxicity in our assay, indicating that there could be a link between regulation of the cell wall and cytotoxicity. Nutrient sensing also appeared to have an impact on cytotoxicity as several TCS mutants (*kdpE::tn*, *phoP::tn* and *hptS::tn*) had reduced cytotoxicity compared to the wild-type strain. In contrast, TCSs involved in respiration had less of an impact on cytotoxicity, with only the *nreC::tn* mutant displaying reduced THP-1 killing. Interestingly, the HK and RR mutants of DesRK, a TCS with uncharacterized function, had reduced cytotoxicity compared to the wild-type strain. Overall, these data demonstrate that the majority of the TCSs in *S. aureus* contribute to regulating cytotoxicity.

### The *phoP::tn* mutant secretes a lower abundance of PSMs

**Fig. 2.**
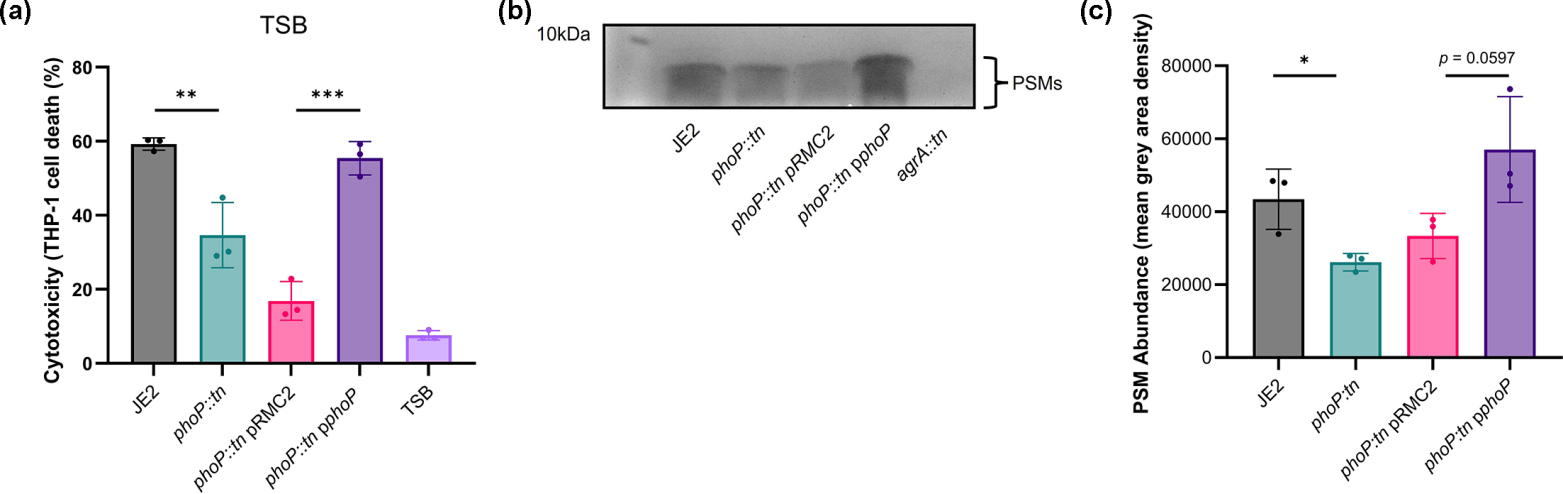
The *phoP::tn* mutant has a lower abundance of PSMs in the supernatant. (**a**) THP-1 cell lysis upon incubation with bacterial supernatant was quantified using trypan blue exclusion. The *phoP::tn* mutant exhibits lower cytotoxicity to THP-1 cells, an effect which can be complemented by expressing *phoP* from the pRMC2 plasmid. (b, c) Bacterial supernatants were extracted and separated via SDS-PAGE, and protein bands were visualized using Coomassie staining. (**b**) A representative SDS-PAGE gel showing PSMs in strains, which are visualized just below the 10 kDa molecular weight marker, as shown previously [69,71]. The band is absent in an *agrA::tn* mutant, which does not produce PSMs, and a lower abundance can be seen in the *phoP::tn* mutant, which can be complemented. (**c**) Mean grey area of PSM bands calculated using densitometry. Significance in (a, c) is denoted as *P<0.05, **P<0.01 and ***P<0.001.

We focused on the *phoP::tn* mutant because the PhoPR signalling mechanism is well characterized in *S. aureus* and other bacteria, yet its role in cytotoxicity and broader gene regulation remains unexplored [37,40]. Notably, our screen showed that only *phoP*, not its cognate kinase *phoR*, impacted cytotoxicity in TSB, suggesting that PhoP may modulate virulence independently of PhoR. Overall, this made it a compelling candidate to pursue for novel insights into cytotoxicity regulation. PhoPR is a Pi-responsive TCS which is present in many bacterial species [40]. PhoR, the HK of the system, undergoes autophosphorylation in response to Pi limitation and then phosphorylates PhoP, the RR, which can activate or repress target genes [40,41]. Whilst most of these genes are involved in phosphate homeostasis, the PhoPR TCS has been linked to regulating virulence factors in several other bacterial species such as *E. coli*, *Mycobacterium tuberculosis* and *Corynebacterium glutamicum* [42,45]. Therefore, we wanted to establish whether phosphate sensing regulates cytotoxicity in *S. aureus*. Firstly, we sought to rule out any polar effects of the transposon insertion or spurious effects of mutations elsewhere on the chromosome. Therefore, we complemented the *phoP* mutation using the pRMC2 vector (pRMC2::*phoP*). Expression of *phoP* in the *phoP*::tn mutant restored cytotoxicity to wild-type levels (Fig. 2a). As THP-1 cells are particularly sensitive to lysis by PSMs, due to their potent, receptor-independent membrane-disrupting activity, we hypothesized that PSM abundance may be reduced in the supernatant of the *phoP::tn* mutant [46,47]. To test this, we extracted the bacterial supernatant and analysed PSM abundance using SDS-PAGE (Fig. 2b, cropped version of replicate 1). Densitometry was performed on three biological replicates (Fig. 2b), and uncropped versions of these are available in Fig. S1. All strains reached an equivalent OD (OD_600_) prior to extraction of supernatants. An *agrA::tn* mutant, which does not produce any PSMs, was used as a control. We found that the *phoP::tn* mutant has a lower abundance of PSMs in the supernatant compared to the wild-type strain, explaining the reduced cytotoxicity of this strain. Additionally, this phenotype could also be complemented by expressing *phoP* from the pRMC2 vector.

### The *phoP::tn* mutant has reduced Agr activity

**Fig. 3.**
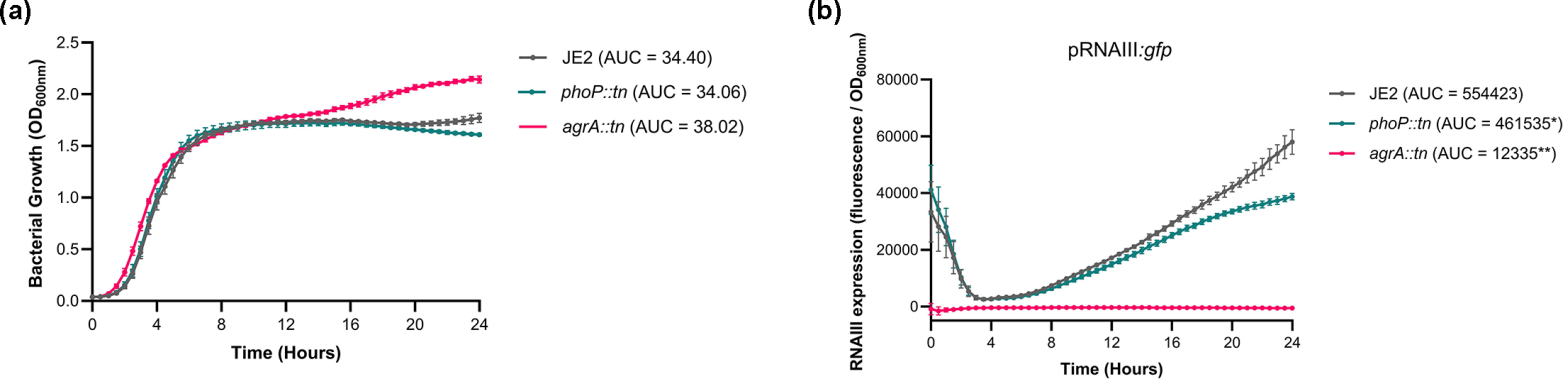
Activity of the Agr system is reduced in the *phoP::tn* mutant. (**a**) Growth of JE2 and the *phoP::tn* mutant carrying an RNAIII::*gfp* fusion plasmid was measured over 24 h, demonstrating that mutation of *phoP* has no effect on *S. aureus* growth. An *agrA::tn* mutant was used as a control. (**b**) Fluorescence (F) of JE2 and the *phoP::tn* mutant was measured to quantify activation of the Agr system. The *phoP::tn* mutant exhibited reduced fluorescence over 24 h, indicating that this mutant has impaired Agr activity. Area under the curve (AUC) analysis was performed on OD_600nm_ and F/OD values, and significance was determined using a t-test. Significance is denoted as *P<0.01 and **P<0.0001.

Given that the Agr TCS is known to directly regulate expression of PSMs [22], we hypothesized that the activity of the Agr system was reduced in the *phoP::tn* mutant. To test this, we introduced an RNAIII::*gfp* fusion plasmid, which acts as a reporter of Agr activity, into the wild-type strain and *phoP::tn* mutant and measured fluorescence and growth over a 24 h period (Fig. 3a, b). An *agrA::tn* mutant, which has no Agr activity, was used as a control. Despite minor differences in growth, there was a significant reduction in fluorescence in the *phoP::tn* mutant, demonstrating that RNAIII transcription was reduced in comparison to the wild-type strain. The modest reduction in Agr activity observed in the *phoP::tn* mutant correlated with the extent of the cytotoxicity defect in the mutant, supporting the biological relevance of this effect. As RNAIII encodes for Hld, a PSM, and regulates expression of other toxins, this explains why PSM abundance and cytotoxicity are reduced in the *phoP::tn* mutant.

### PhoP differentially regulates Agr activity depending on phosphate availability

**Fig. 4.**
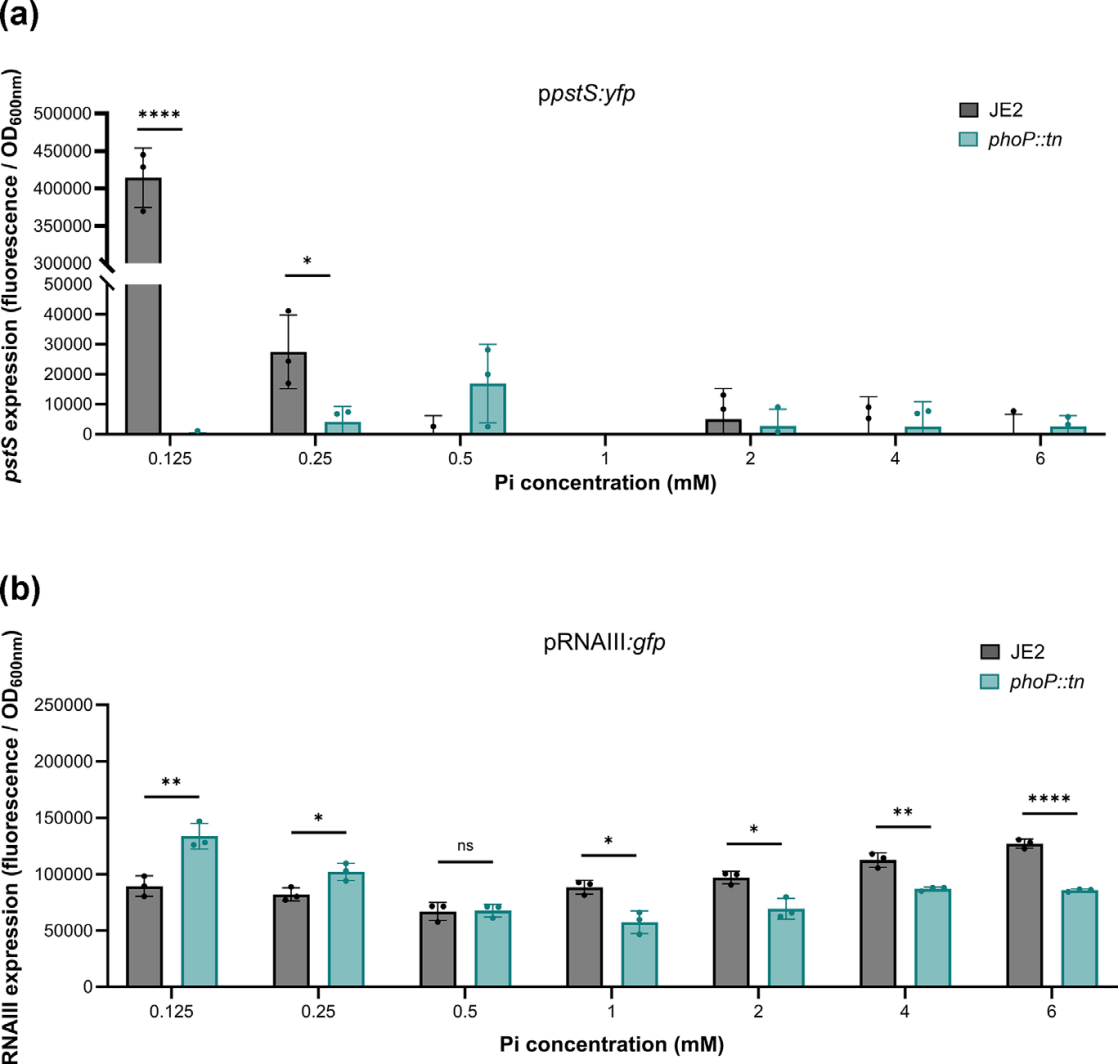
PhoP differentially regulates Agr activity depending on phosphate availability. (**a**) Fluorescence (F) of JE2 WT and *phoP::tn* mutant carrying a *pstS::yfp* reporter plasmid was measured following growth in phosphate-depleted RPMI media supplemented with a range of Pi concentrations to identify PhoPR activation. Expression of *pstS* occurs below 0.25 mM Pi, and this induction does not occur in the *phoP::tn* mutant. (**b**) Fluorescence of JE2 and the *phoP::tn* mutant carrying an RNAIII::*gfp* reporter plasmid was measured in the same condition to determine Agr activity. The *phoP::tn* mutant had reduced fluorescence at high Pi concentrations, but higher fluorescence under Pi limitation compared to the wild-type strain. Each dot represents one biological replicate (*n* = 3), error bars represent the sd and statistical significance was determined using a *t*-test. Significance is denoted as *P<0.05, **P< 0.01 and ***P<0.001.

As PhoPR is activated by Pi limitation, we wanted to determine whether PhoP alters Agr activity in response to Pi availability. Induction of *pstS* expression, a phosphate-binding protein in the *pstSCAB* operon, can be used as a marker of PhoPR activation and Pi limitation, as *pstS* expression is induced by phosphorylated PhoP [17,48]. To establish a model of Pi limitation, we introduced a *pstS::yfp* reporter fusion plasmid into the wild-type strain and *phoP::tn* mutant. We then compared fluorescence of the wild-type strain and *phoP*::tn mutant following an overnight growth in phosphate-depleted RPMI media supplemented with a range of Pi concentrations (Fig. 4a). In all Pi concentrations we tested (ranging from 0.125 to 6 mM), the wild-type and *phoP::tn* mutant grew to a similar OD_600nm_ (Fig. S2a, b). We detected *pstS* induction in the wild-type strain when Pi availability was ≤0.25 mM. In contrast, *pstS* induction was absent in the *phoP::tn* mutant, indicating specific regulation of this gene by PhoP.

Next, we measured Agr activity in the same growth conditions. Fluorescence of an *agrA::tn* mutant was also measured as a control in this experiment; however, these data are not included in Fig. 4(b), as the expression values are zero. In both the wild-type strain and *phoP::tn* mutant, fluorescence was detected in all conditions, demonstrating that the Agr system maintains activity in all Pi concentrations and with disruption of *phoP*. However, when Pi availability was relatively high (between 1 and 6 mM Pi), the *phoP::tn* mutant had significantly reduced Agr activity compared to the wild-type strain. This was similar to what was observed in TSB (Fig. 3b), which is a phosphate-rich medium (~14 mM Pi). When Pi availability was ≤0.25 mM, we found that the *phoP::tn* mutant had significantly higher Agr activity compared to the wild-type strain. Furthermore, Agr activity increased in the WT strain between 0.125 and 6 mM (*P*=0.0027), further supporting the notion that Pi availability regulates Agr activity. Based on the characterization of the PhoRP signalling mechanism in other bacteria where it has been demonstrated that low Pi results in phosphorylation of PhoP by PhoR, our data support a model whereby PhoP exhibits differential regulation of Agr activity depending on Pi availability and its phosphorylation state [37,40, 41, 49]. When Pi availability is high, PhoP is unphosphorylated and upregulates Agr activity. In contrast, when Pi availability is low and PhoP is phosphorylated, it represses Agr activity. THP-1 cytotoxicity data for the wild-type and *phoP::tn* mutant in these growth conditions are provided in Fig. S3.

### PhoP represses cytotoxicity in low-Pi environments

**Fig. 5.**
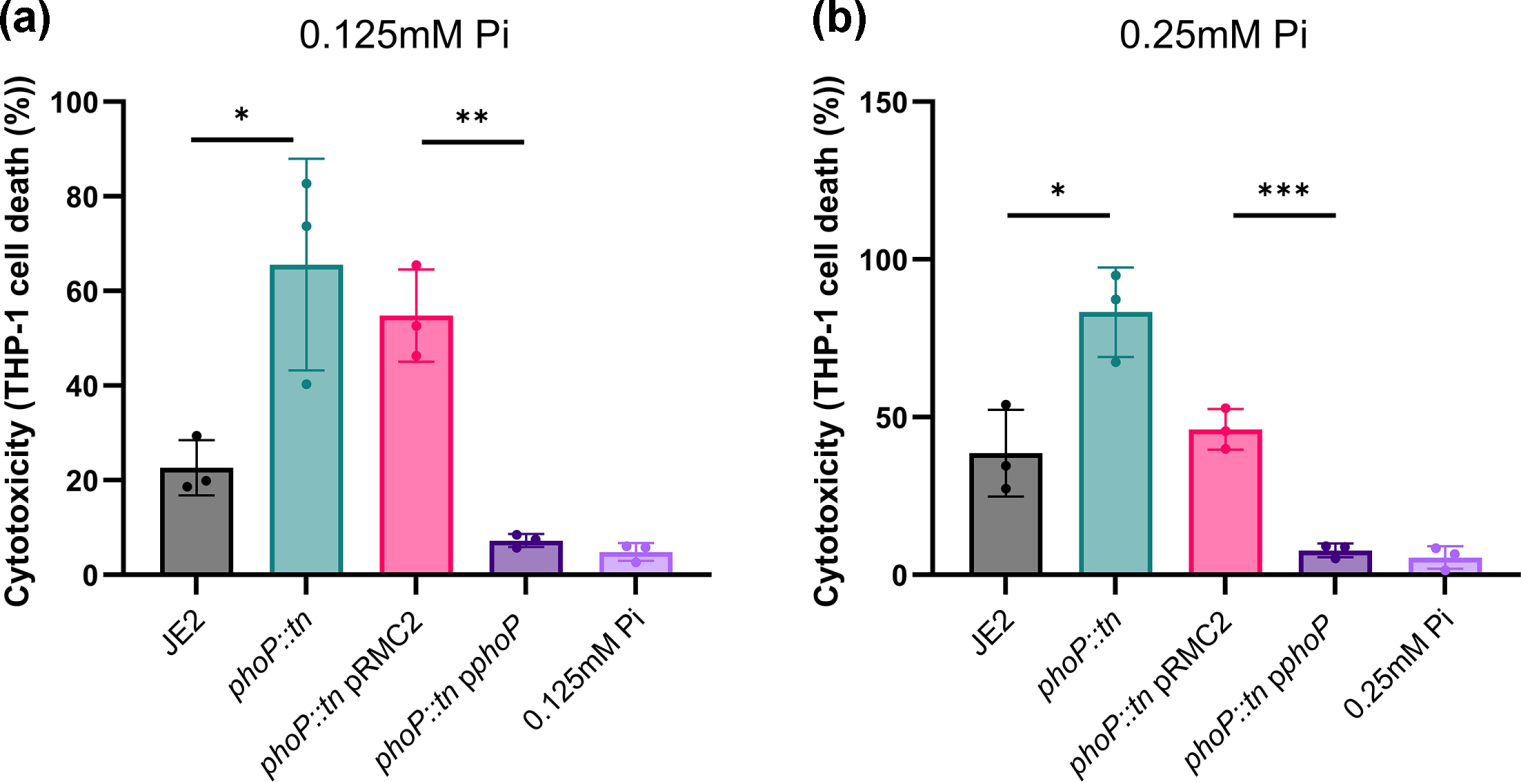
PhoP acts as a repressor of cytotoxicity in low-Pi environments. (a, b) THP-1 cell lysis upon incubation with bacterial supernatant was quantified using trypan blue exclusion. Supernatants were extracted following growth of strains in phosphate-depleted RPMI media supplemented with either 0.125 mM Pi (**a**) or 0.25 mM Pi (**b**). In both conditions, the *phoP::tn* mutant has higher cytotoxicity compared to the wild-type strain, and expression of phoP from the pRMC2 significantly represses cytotoxicity compared to the *phoP::tn* mutant complemented with an empty pRMC2 vector. Significance is denoted as *P<0.05, **P< 0.01 and ***P<0.001.

Our data demonstrate that PhoP acts as an activator in high-Pi environments, as the *phoP::tn* mutant has lower Agr activity and cytotoxicity, and overexpression of *phoP* increases cytotoxicity in these growth conditions. In contrast, we found that the *phoP::tn* mutant has higher Agr activity in low-Pi environments (≤0.25 mM), suggesting that it may act as a repressor in this environment. To confirm this, we tested the cytotoxicity of strains following growth in either 0.125 mM or 0.25 mM Pi (Fig. 5a, b). In line with Agr activity under low-Pi conditions, the *phoP::tn* mutant had significantly higher cytotoxicity compared to the wild-type strain in both 0.125 mM or 0.25 mM Pi. Furthermore, expression of *phoP* in the *phoP*::tn mutant significantly repressed cytotoxicity, confirming that PhoP acts as a cytotoxicity repressor in low Pi environments.

### Regulation of Agr activity by PhoP is determined by its phosphorylation state

**Fig. 6.**
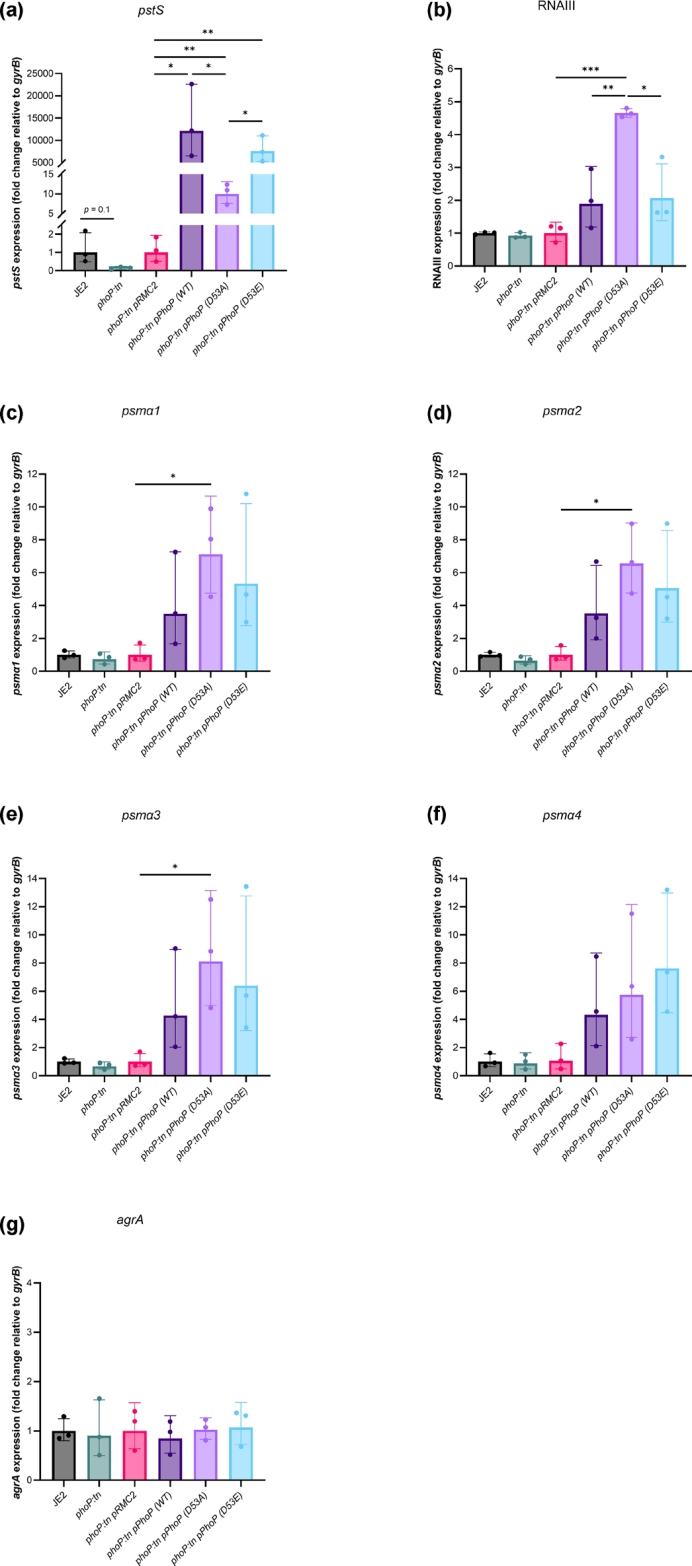
The phosphorylation state of PhoP determines Agr regulation. Gene expression of *pstS* (**a**), RNAIII (**b**), *psmα1* (**c**), *psmα2* (**d**), *psmα3* (**e**), *psmα4* (**f**) and *agrA* (**g**) was quantified relative to expression levels of the *gyrB* housekeeping gene in JE2 (wild type), the *phoP::tn* mutant and strains carrying the empty pRMC2 vector, WT, D53A or D53E PhoP following induction with aTC. Fold change was then calculated relative to the expression of each target gene to either the wild-type strain or the *phoP::tn* mutant carrying the empty pRMC2 vector. Expression of *pstS* was significantly higher upon expression of WT or D53E PhoP compared to D53A PhoP. In contrast, expression of D53A PhoP resulted in higher levels of RNAIII, *psmα1*, *psmα2*, *psmα3* and *psmα4* compared to WT and D53E PhoP. No difference in *agrA* expression was observed in any of the strains. Each dot represents one biological replicate (*n* = 3), error bars represents the sd and statistical significance was determined by a t-test. Significance is denoted as *P<0.05, **P<0.01 and ***P<0.001.

As we identified that PhoP can act as either an activator or repressor of cytotoxicity depending on Pi availability, we wanted to verify the impact of PhoP phosphorylation on Agr activity. To test this, we substituted the phosphorylation site of PhoP (D53) in pRMC2::*phoP* with an alanine, which prevents phosphorylation (phosphoablative), or a glutamic acid residue, which mimics the conformational change induced by permanent phosphorylation of the PhoP protein (phosphomimetic) [17]. We then induced expression of WT, D53A or D53E PhoP with the addition of aTC to growth media (TSB) and measured expression of *pstS*, RNAIII, *psmα1*, *psmα2*, *psmα3*, *psmα4* and *agrA* relative to a control strain carrying the empty pRMC2 vector. Expression was also measured and compared between JE2 and the *phoP::tn* mutant (Fig. 6a–f) in TSB. As the pRMC2 and pRNAIII::*gfp* vectors use the same resistance marker, we measured the levels of *pstS* and RNAIII using qRT-PCR. Inductions were performed in TSB, a high-Pi medium.

Transcription of *pstS* was consistently lower in the *phoP::tn* mutant compared to the wild-type strain, although this did not reach statistical significance. However, transcription of *pstS* was significantly elevated in *phoP:tn* expressing either WT, D53A or D53E PhoP compared to *phoP:tn* pRMC2. This was ~1,000-fold higher upon expression of either WT or D53E PhoP compared to D53A PhoP, suggesting that induction of *pstS* expression is mediated by WT or phosphorylated PhoP. Transcription of RNAIII was also elevated in all strains. However, expression of D53A PhoP resulted in approximately twofold higher RNAIII transcription compared to WT and D53E PhoP. No difference in RNAIII transcription was observed between the *phoP::tn* mutant compared to the wild-type strain. Slightly reduced levels of *psmα1*, *psmα2* and *psmα3* were also observed in the *phoP::tn* mutant compared to the wild-type strain, although this also did not reach statistical significance. However, expression of WT, D53A and D53E PhoP increased expression of *psmα1*, *psmα2*, *psmα3* and *psmα4*, but expression of D53A PhoP resulted in the highest levels of induction. Interestingly, no differences in *agrA* expression were observed in any of the strains. Combined, these data confirm that phosphorylation state can affect PhoP regulation of cytotoxicity and suggest that regulation of *α*-type PSMs may also contribute to the cytotoxicity defect and reduced PSM abundance in the *phoP:tn* mutant.

## Discussion

In this study, we systematically compared the contribution to cytotoxicity of all the non-essential TCSs in the genome of *S. aureus* strain JE2. Our screening identified a link between TCSs involved in cell wall biosynthesis or autolysis and regulation of cytotoxicity as several mutants (*graR::tn*, *graS::tn*, *nsaR::tn* and *lytR::tn*) had reduced THP-1 toxicity. Disruption of these TCSs has profound effects on cell surface properties and morphology [18,50,53]. Accordingly, changes to the cell surface structure of *S. aureus* have been previously reported to affect the regulation and secretion of toxins [38,54]. Nutrient sensing was also identified as an important signal for regulating cytotoxicity, with *kdpE::tn*, *phoP::tn* and *hptS::tn* mutants exhibiting reduced cytotoxicity. As the biosynthesis of cytolytic toxins is an energetically costly process, many pathogenic bacteria synchronize this with nutrient availability [14,55]. Furthermore, nutrient availability can vary between different host tissues, acting as a marker for host environments where cytotoxicity is an advantageous phenotype.

**Fig. 7.**
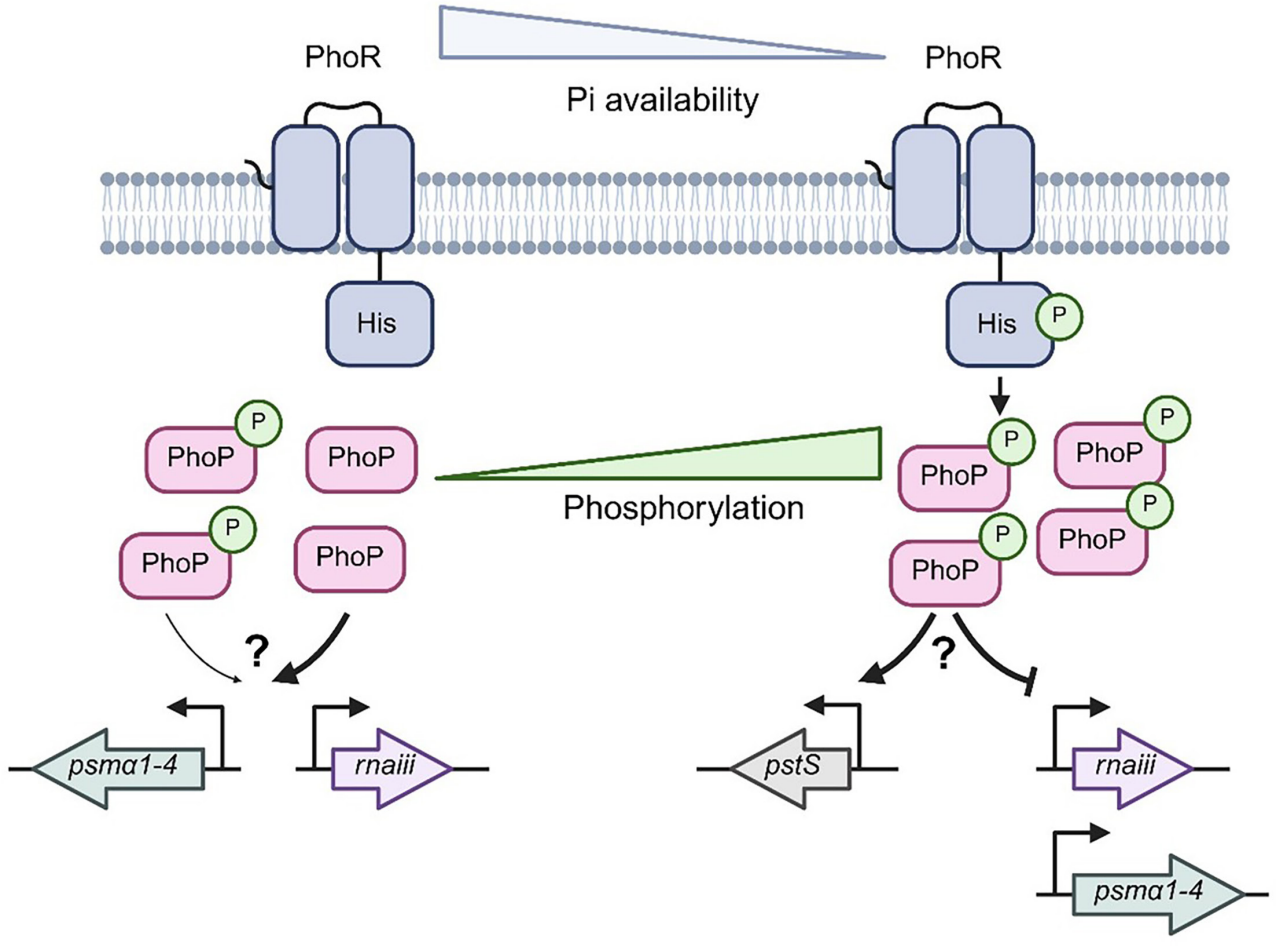
Summary of Agr regulation by PhoP. When phosphate (Pi) availability is high, PhoR is inactive, and PhoP mainly exists in its unphosphorylated form. Unphosphorylated PhoP acts as a strong activator of RNAIII via the P3 promoter and *psmα1–4* expression. Phosphorylated PhoP also acts as a weaker activator of RNAIII and *psmα1–4*. When Pi availability decreases, activation of PhoR occurs. PhoR undergoes autophosphorylation and mediates phosphorylation of PhoP. Phosphorylated PhoP induces expression of genes involved in phosphate transport, including *pstS*. Furthermore, phosphorylated PhoP acts as a repressor of cytotoxicity in low-Pi environments, likely through regulation of RNAIII and *psmα1–4*. Question marks are used to indicate outstanding questions about this regulatory network. Namely, further investigation is required to understand if regulation of RNAIII and *psmα1–4* is direct or indirect. Furthermore, we do not yet understand why phosphorylated PhoP switches from being an activator to a repressor of cytotoxicity from high Pi to low Pi.

Having decided to focus here on the effect of PhoPR on cytotoxicity, we demonstrated that the *phoP::tn* mutant produces less PSMs in the bacterial supernatant during growth in TSB (summarized in Fig. 7). PSMs include PSMα1–4 and PSMβ1/2, which are directly regulated by phosphorylated AgrA. Additionally, Hld is encoded within the main effector molecule of the Agr system, RNAIII [56,57]. As PSMβ1/2 are non-cytolytic towards eukaryotic cells, we have focused on Hld and PSMα1–4 [47]. We identified that the *phoP::tn* mutant has lower RNAIII expression, suggesting that this mutant has reduced amounts of Hld and lower Agr activity. This finding supports the notion that TCSs in *S. aureus* are a highly interconnected network which frequently interacts with each other to fine-tune gene expression [3,31]. For example, expression of a phosphomimetic form of WalR activates the Sae TCS [30]. Similarly, glucose-6-phosphate induces expression of both Agr and Sae, and this is dependent on the presence of the HptRS TCS [58]. Interestingly, overexpression of D53A PhoP, and to a lesser extent WT/D53E PhoP, increased transcription of *psmα1–4*, and transcription of *psmα1*, *psmα2 and psmα3* was moderately decreased in the *phoP::tn* mutant. Therefore, regulatory control of these genes by PhoP may also contribute to the reduced cytotoxicity of the *phoP::tn* mutant.

Induction of *pstS* by PhoP follows the canonical signalling mechanism by which a TCS activates a target gene. For example, we detected induction of *pstS* expression in growth media≤0.25 mM Pi, where PhoPR is activated and levels of phosphorylated PhoP significantly increase [17,48]. In contrast, no regulation of *pstS* was detected when Pi levels were ≥0.5 mM, where PhoP is unphosphorylated. This mechanism was confirmed through expression of D53E PhoP, which resulted in 1,146-fold higher levels of *pstS* compared to expression of D53A PhoP. However, PhoP-mediated regulation of cytotoxicity follows an atypical trend. Firstly, unphosphorylated PhoP acts as a strong activator of RNAIII and *psmα1–4* transcription in high-Pi environments. This allows for regulation of cytotoxicity in the absence of an activation signal and explains why a *phoR::tn* mutant did not show a reduction in cytotoxicity in our initial screening as the experiment was performed at high Pi. Secondly, we identified that phosphorylated PhoP has differential effects on cytotoxicity depending on Pi concentration, acting as a weak activator in high Pi and a repressor in low Pi. Our current hypothesis is that additional factors expressed at low Pi regulated by PhoP concur in the regulation of cytotoxicity. This would explain why RNAIII transcription and cytotoxicity are elevated in a *phoP::tn* under Pi limitation, and overexpression of PhoP in this condition strongly represses cytotoxicity. This also suggests that regulation of cytotoxicity by Pi is a multifactorial process, with additional factors involved in coordination with PhoPR.

We do not yet understand whether the regulatory control of PhoP over RNAIII and *psmα1–4* occurs via direct binding to their respective promoters or indirectly through other regulators. Interestingly, *agrA* expression was unaffected in the *phoP::tn* mutant or by overexpression of PhoP. This suggests that PhoP only regulates the P3 promoter of the Agr system (RNAIII) and that regulation of *psmα1–4* may occur in an AgrA-independent mechanism. The consensus binding sequence of PhoP is known in *Bacillus subtilis* as TT(A/T/C)ACA-N_4–6_-TT(A/T/C)ACA [59]. In the USA300_FPR3757 reference genome, this sequence is also present in the promoter for *psmα1–4*, and its reverse complement is present upstream of *pstS*. No binding sequence was found in the P3 promoter for RNAIII. Therefore, we hypothesize that regulation of *psmα1–4* by PhoP may occur via direct binding, but confirmation of this is currently under investigation.

The finding that unphosphorylated PhoP is a stronger activator of RNAIII and *psmα1–4* than phosphorylated PhoP in high-Pi environments adds to growing experimental evidence which indicates that unphosphorylated RRs have important regulatory functions [60]. For example, unphosphorylated WalR from *Streptococcus pneumoniae* inhibits *fabT*, a repressor of fatty acid chain elongation [61]. This supports the idea that TCSs can initiate a more complex response than a simple on–off feed-forward mechanism, providing several regulatory options depending on their phosphorylation status. One more potential layer of regulation could be represented by the expression levels of PhoP, which may also change depending on Pi availability and affect how PhoPR regulates Agr activity.

To date, studies of PhoPR in *S. aureus* have focused on its role in regulating phosphate transporters (*pstSCAB*, *nptA* and *pitA*) in response to phosphate limitation [48]. *S. aureus* encounters a range of Pi concentrations during colonization and infection [62]. For example, Pi concentrations are low in sweat (~0.057 mM) and blood (~0.38 mM) and much higher in saliva (~28–36 mM) and nasal secretions (ranging from 0.75 to 7 mM) [63,66]. Importantly, an *S. aureus* Δ*phoPR* mutant was found to be attenuated in the heart during a systemic staphylococcal abscess model of infection, whereas a Δ*pstSCAB* Δ*nptA* double mutant had no defect, suggesting that transporter-independent factors contribute to *S. aureus* pathogenesis in this environment [48]. Our findings that PhoPR regulates cytotoxicity provide a potential explanation for this virulence defect and suggest that this regulation may be relevant during *in vivo* infections. All experiments were performed in *S. aureus* JE2 as this is the wild-type strain of the NTML from which the TCS mutants were sourced. Whilst this is derived from a clinically relevant CA-MRSA strain, regulatory mechanisms can differ between *S. aureus* strains. Therefore, exploring the regulation of cytotoxicity by PhoPR in additional strains represents a valuable avenue for future research.

In conclusion, our systematic screening approach revealed how the regulatory effects of 11 out of 16 TCSs converge on cytotoxicity. Our results confirm that virulence regulation depends on an intricate regulatory network that reacts to diverse environmental conditions. We identified phosphate sensing via PhoP as a novel regulator of cytotoxicity in *S. aureus*. PhoP interacts with the Agr system to regulate expression of PSMs. Crucially, PhoP mediates differential regulation of RNAIII expression depending on environmental Pi and the balance between its unphosphorylated and phosphorylated forms. This work builds upon previous studies which have identified the PhoPR TCS as important for the full virulence of *S. aureus*, and inhibition of this system represents a promising therapeutic target.

## Supplementary material

10.1099/mic.0.001606 Uncited Fig. S1.

## Abbreviations

Agr: accessory gene regulator
Arl: autolysis-related locus
aTC: anhydrotetracycline
FDR: false discovery rate
HK: histidine kinase
Hla: alpha-haemolysin
NTML: Nebraska Transposon Mutant Library
Pi: inorganic phosphate
PSMs: phenol-soluble modulins
qRT-PCR: quantitative reverse transcription PCR
RR: response regulator
Sae: staphylococcal accessory element
TCSs: two-component systems
TSA: tryptic soy agar
TSB: tryptic soy broth
