# Supplementary material for: Phosphate sensing by PhoPR regulates the cytotoxicity of Staphylococcus aureus
Source: Microbiology (Reading). 2025 Sep 2;171(9):001606. doi: 10.1099/mic.0.001606 (PMC12404567; doi:10.1099/mic.0.001606)
Supplement: Uncited Fig. S1. [file mic-171-01606-s001.pdf]

Supplementary Figures

Strain	*p*-value	Adjusted *p*-value
agrA	0.000004	0.000041
agrC	<0.000001	0.000009
saeR	0.000391	0.001579
saeS	0.000805	0.002324
arlR	0.004718	0.007340
arlS	0.000292	0.001475
graR	0.009111	0.010826
graS	0.006006	0.008666
vraR	0.038405	0.043099
vraS	0.106537	0.097820
nsaR	0.000769	0.002324
nsaS	0.000077	0.000518
lytR	0.003554	0.007180
lytS	0.061038	0.064893
kdpE	0.004303	0.007340
kdpD	0.191049	0.160800
phoP	0.001853	0.004423
phoR	0.077734	0.078511
hptR	0.227762	0.184032
hptS	0.001971	0.004423
hssR	0.124835	0.109638
hssS	0.411666	0.307987
srrA	0.639063	0.461038
srrB	0.783256	0.545578
nreC	0.004723	0.007340
nreB	0.381898	0.296706
airS	0.101433	0.097569
desR	0.007443	0.009396
desK	0.006940	0.009346

Supplementary Table 1. Statistical analysis of cytolytic activity in TCS transposon mutants. Significance was determined using multiple t-tests with a false discovery rate (FDR) of 1% applied. Differences in cytotoxicity were considered significant if they had an adjusted p-value ≤0.01.

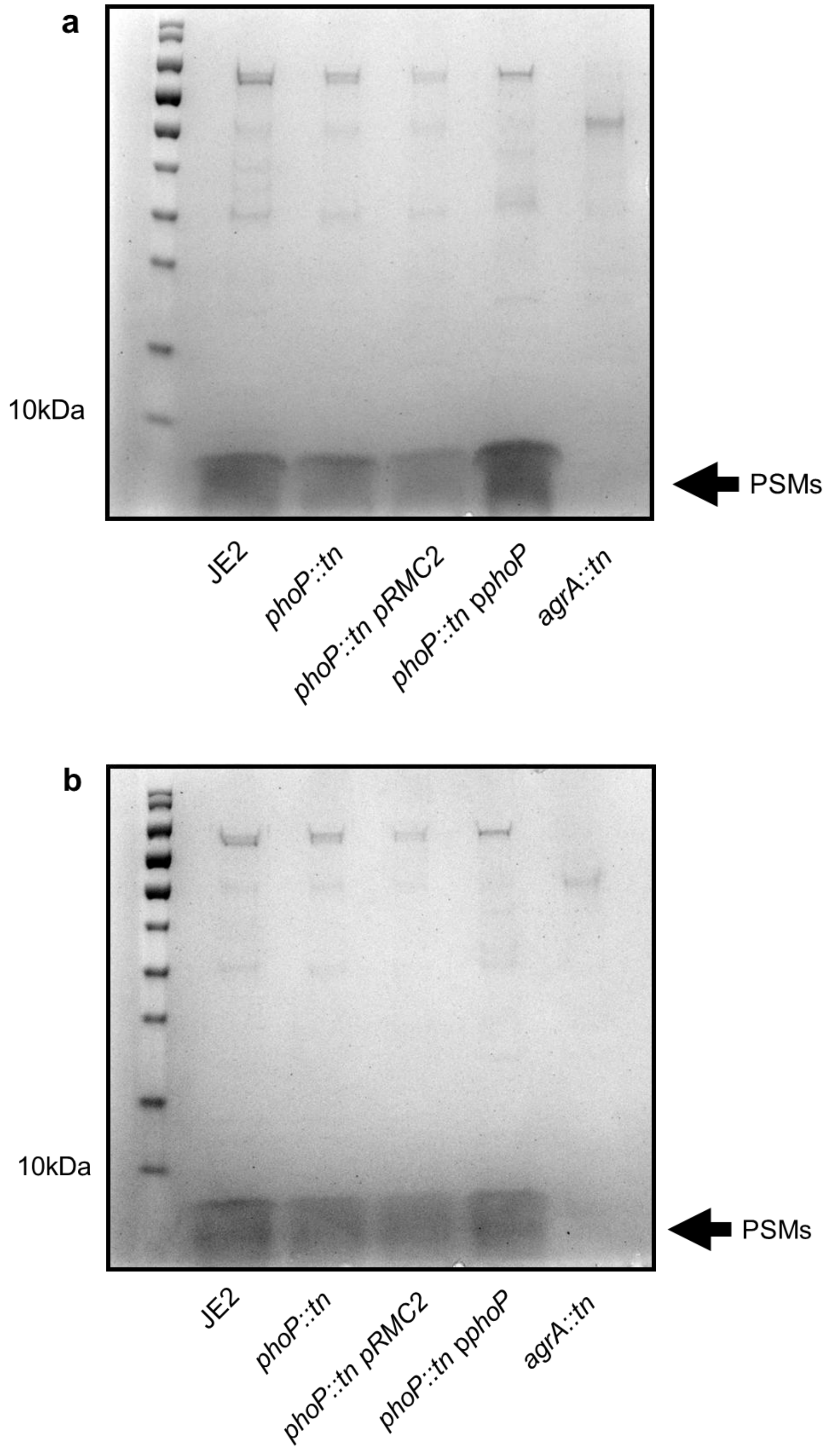
a
10kDa
PSMs
JE2
phoP::tn
phoP::tn pRMC2
phoP::tn pphoP
agrA::tn
b
10kDa
PSMs
JE2
phoP::tn
phoP::tn pRMC2
phoP::tn pphoP
agrA::tn

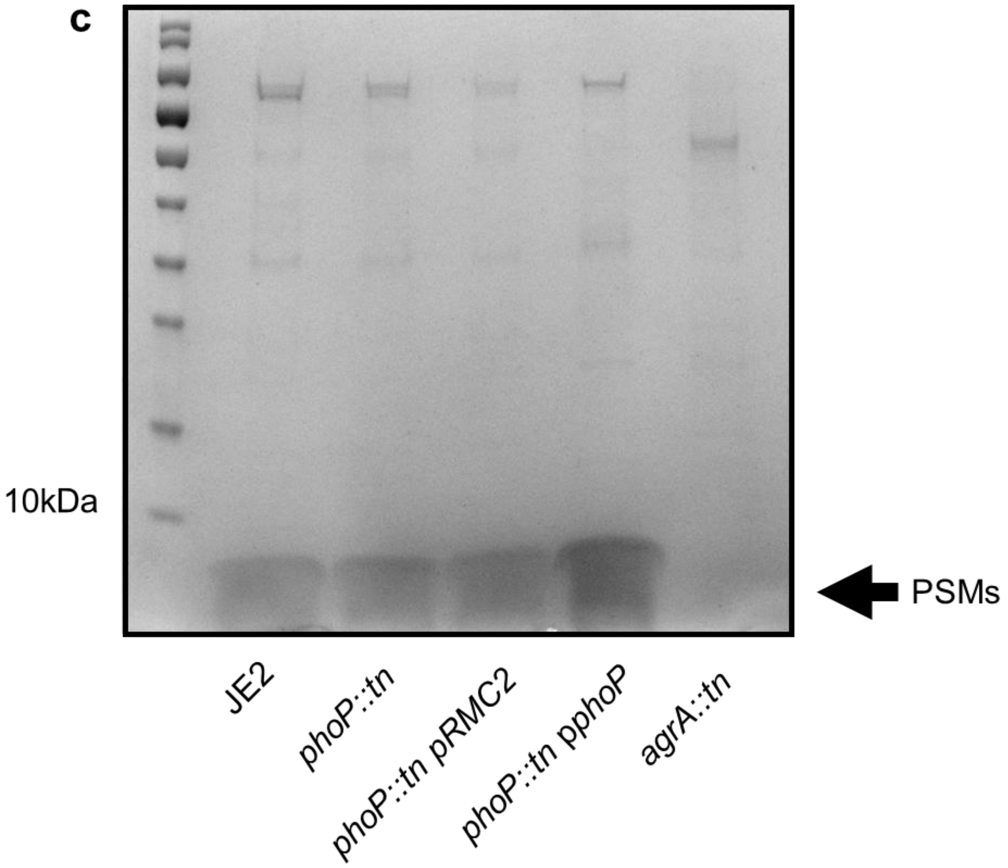

Supplementary Figure 1. Three replicates (a,b and c) of SDS-PAGE gels showing a reduction in PSMs in the supernatant of the *phoP::tn* mutant, which can be complemented by reintroducing expression of *phoP*.

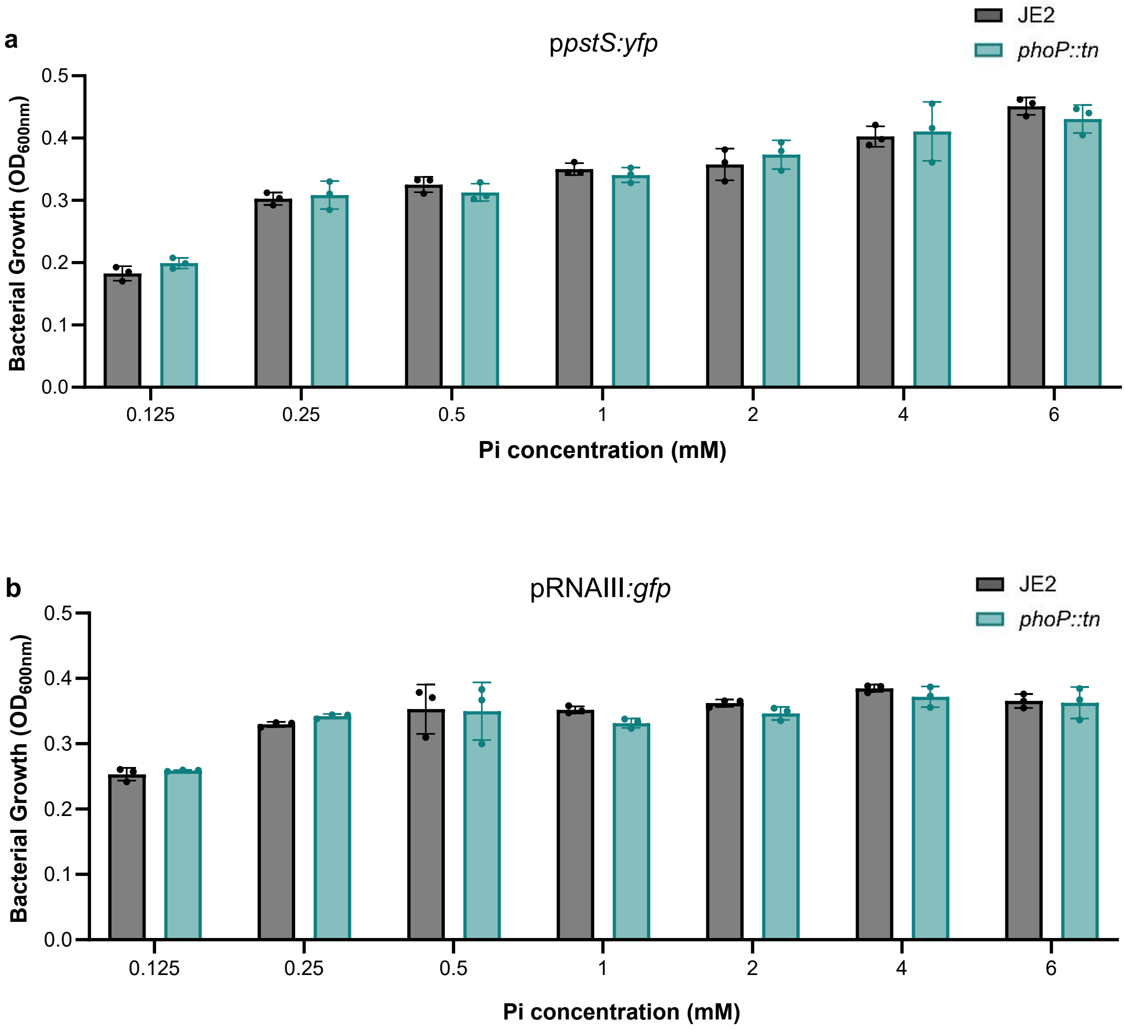

Supplementary Figure 2. Growth of JE2, the *phoP::tn* mutant carrying *ppstS::yfp* (a) or p*rnaiii::gfp* (b) was measured from an overnight culture in RPMI media with different phosphate concentrations. All three strains grew to a similar OD_{600nm} in all concentrations tested.

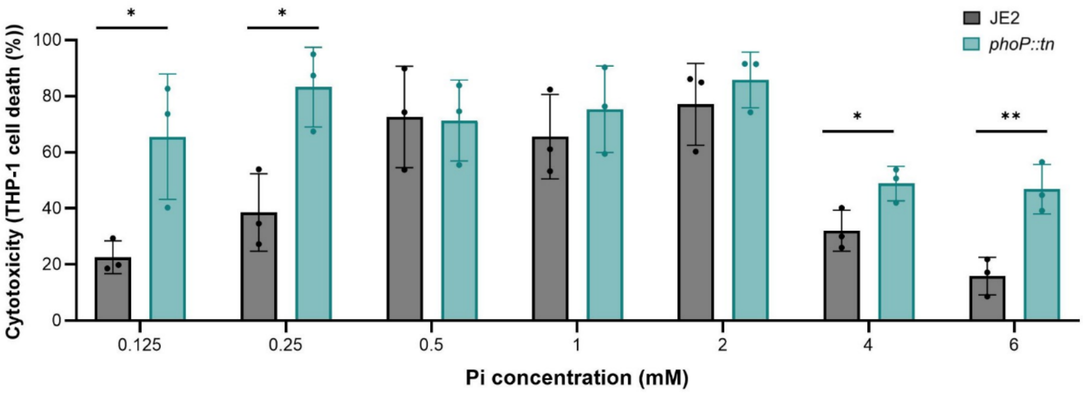

Supplementary Figure 3. THP-1 cytotoxicity JE2, the *phoP::tn* mutant was measured from an overnight culture in RPMI media with different phosphate concentrations. All strains grew to a similar OD_{600nm} in all concentrations tested.
